# The role of vitamin D in sarcoidosis

**DOI:** 10.12703/b/9-14

**Published:** 2020-11-18

**Authors:** Fabiola Gianella, Connie CW Hsia, Khashayar Sakhaee

**Affiliations:** 1 Charles and Jane Pak Center for Mineral Metabolism and Clinical Research, University of Texas Southwestern Medical Center, Dallas, Texas, USA; 2 Pulmonary and Critical Care Medicine Division, University of Texas Southwestern Medical Center, Dallas, Texas, USA; 3 Department of Internal Medicine, University of Texas Southwestern Medical Center, Dallas, Texas, USA

**Keywords:** Sarcoidosis, hypercalcemia, kidney stones, vitamin D, autoimmune

## Abstract

After the initial description of extrarenal synthesis of 1,25-dihydroxyvitamin D (1,25-(OH)_2_D) three decades ago, extensive progress has been made in unraveling the immunomodulatory roles of vitamin D in the pathogenesis of granulomatous disorders, including sarcoidosis. It has been shown that 1,25-(OH)_2_D has dual effects on the immune system, including upregulating innate immunity as well as downregulating the autoimmune response. The latter mechanism plays an important role in the pathogenesis and treatment of sarcoidosis. Vitamin D supplementation in patients with sarcoidosis has been hampered owing to concerns about the development of hypercalcemia and hypercalciuria given that extrarenal 1-α hydroxylase is substrate dependent. Recently, a few studies have cast doubt over the mechanisms underlying the development of hypercalcemia in this population. These studies demonstrated an inverse relationship between the level of vitamin D and severity of sarcoidosis. Consequently, clinical interest has been piqued in the use of vitamin D to attenuate the autoimmune response in this disorder. However, the development of hypercalcemia and the attendant detrimental effects are real possibilities. Although the average serum calcium concentration did not change following vitamin D supplementation, in two recent studies, hypercalciuria occurred in one out of 13 and two out of 16 patients. This review is a concise summary of the literature, outlining past work and newer developments in the use of vitamin D in sarcoidosis. We feel that larger-scale placebo-controlled randomized studies are needed in this population. Since the current first-line treatment of sarcoidosis is glucocorticoids, which confer many systemic adverse effects, and steroid-sparing immunosuppressant treatment options carry additional risks of adverse effects, adjunct management with vitamin D in combination with potent anti-osteoporotic medications could minimize the risk of glucocorticoid-induced osteoporosis and modulate the immune system to attenuate disease activity in sarcoidosis.

## Introduction

Under normal circumstances, renal 1α-hydroxylase converts 25-hydroxyvitamin D (25(OH)D) to 1,25-dihydroxyvitamin D (1,25-(OH)_2_D). Evidence for increased circulating 1,25-(OH)_2_D and dysregulation of calcium metabolism in sarcoidosis was initially described in 1979^1,2^. The underlying mechanisms for hypercalcemia were attributed to increased production and/or decreased catabolism of 1,25-(OH)_2_D. Several studies have indicated enhanced intestinal calcium absorption as the main mechanism for the development of hypercalcemia, hypercalciuria, nephrocalcinosis, and abnormal kidney function^3–6^. A case report of hypercalcemia in an anephric patient with sarcoidosis was the first evidence for extrarenal production of 1,25-(OH)_2_D^7^. Subsequent studies using cultured pulmonary alveolar macrophages and sarcoid lymph node homogenates explored the relationship between 25(OH)D and 1-α hydroxylase gene expression^8–10^. Positive findings in the alveolar macrophages support the extrarenal conversion of 25(OH)D to 1,25-(OH)_2_D in sarcoidosis patients^8–10^. These studies were pivotal in unraveling the immunomodulatory role of 1,25-(OH)_2_D in granulomatous diseases such as sarcoidosis and tuberculosis^11^. Here, we review evidence in two distinct ethnic populations with sarcoidosis; both sarcoidosis and vitamin D deficiency are more common in African Americans (AA) than in Caucasians^12^. Moreover, clinical, biochemical, and mineral metabolic perturbations of sarcoidosis in these two distinct ethnicities were similar. Repletion of vitamin D in a small subset of vitamin D-deficient patients with sarcoidosis was found to be safe, effective, and associated with a fall in surrogate markers of sarcoidosis activity and, interestingly, with a fall in serum 1,25-(OH)_2_D^12^. The underlying mechanism(s) of the observed response to vitamin D repletion in this population and the role of 24-hydroxylase in mediating this response deserve further investigation.

## Epidemiology of sarcoidosis

Sarcoidosis is a multi-system inflammatory disorder associated with widespread granuloma deposition leading to multi-organ impairment. The complex causative factors dictate its heterogeneous presentation^13,14^. Geography, race, age, and gender modulate the presentation of sarcoidosis, and it is most prevalent in the Northern hemisphere and among AA individuals^15–17^. Studies in Northern Europe and Japan have described a bimodal age-specific incidence rate among women, with a first peak between 30 and 35 years of age and a second peak over 50 years of age^18–20^.

The incidence and prevalence of sarcoidosis was greater in females than in males in most but not all studies^21,22^. One study in Switzerland found no gender differences in incidence and prevalence of sarcoidosis^23^, while another study in Sweden found a higher incidence in men than in women^15^. The incidence of sarcoidosis in men peaks before 40 years of age; the incidence in women remains flat between the ages of 30 and 60 years^24,25^. The higher prevalence and severity of sarcoidosis among the AA population was purported to be associated with lower vitamin D stores in this group^26–28^. This notion was based on the immunomodulatory effects of vitamin D on the adaptive immune system, which is responsible for the suppression of granulomatous inflammation^29^. However, our study in Dallas^12^ comprising a total of 86 patients with sarcoidosis from two separate ethnic groups from the United States (93% AA) and Italy (95% Caucasian) showed no difference in the clinical, biochemical, vitamin D, and mineral profiles in these two distinct populations^12^.

## Renal synthesis and classical action of 1,25-(OH)_2_D

Vitamin D_3_ (cholecalciferol) is synthesized from exposure to ultraviolet radiation transforming 7-dehydrocholestrol in the skin to vitamin D_3_^30–32^. In addition, vitamin D_3_ is found in the circulation following the consumption of fortified dairy products, orange juice, and fish. Vitamin D_2_ (ergocalciferol) is also synthesized from ergosterol by exposure to ultraviolet radiation. Vitamin D_2_ is found naturally in mushrooms^33^, vitamin D supplements, and with food fortification. Nonetheless, both vitamin D_2_ and vitamin D_3_ undergo the same metabolic pathway.

Circulating vitamin D is initially hydroxylated by three different 25-hydroxylase enzymes (CYP27A1, CYP2R1, and CYP3A4)^34^ in the liver, producing 25(OH)D. 25(OH)D undergoes another hydroxylation by 25(OH)D-1-α hydroxylase (CYP27B1) in the kidney, resulting in the production of 1,25-(OH)_2_D, the most active vitamin D metabolite^35^. 25(OH)D and 1,25(OH)_2_D also undergo 24-hydroxylation by 24-hydroxylase (CYP24A1) to produce inactive metabolites^35^ 24,25-(OH)_2_D and 1,24,25-trihydroxyvitamin D^35^, respectively. The activity of renal CYP27B1 is tightly regulated. Parathyroid hormone (PTH) and hypophosphatemia stimulate 25(OH)D-1-α hydroxylase (CYP27B1) activity^36–39^. Fibroblast growth factor 23 (FGF23) decreases CYP27B1 activity and stimulates CYP24A1 action^40^.

**Figure 1.**
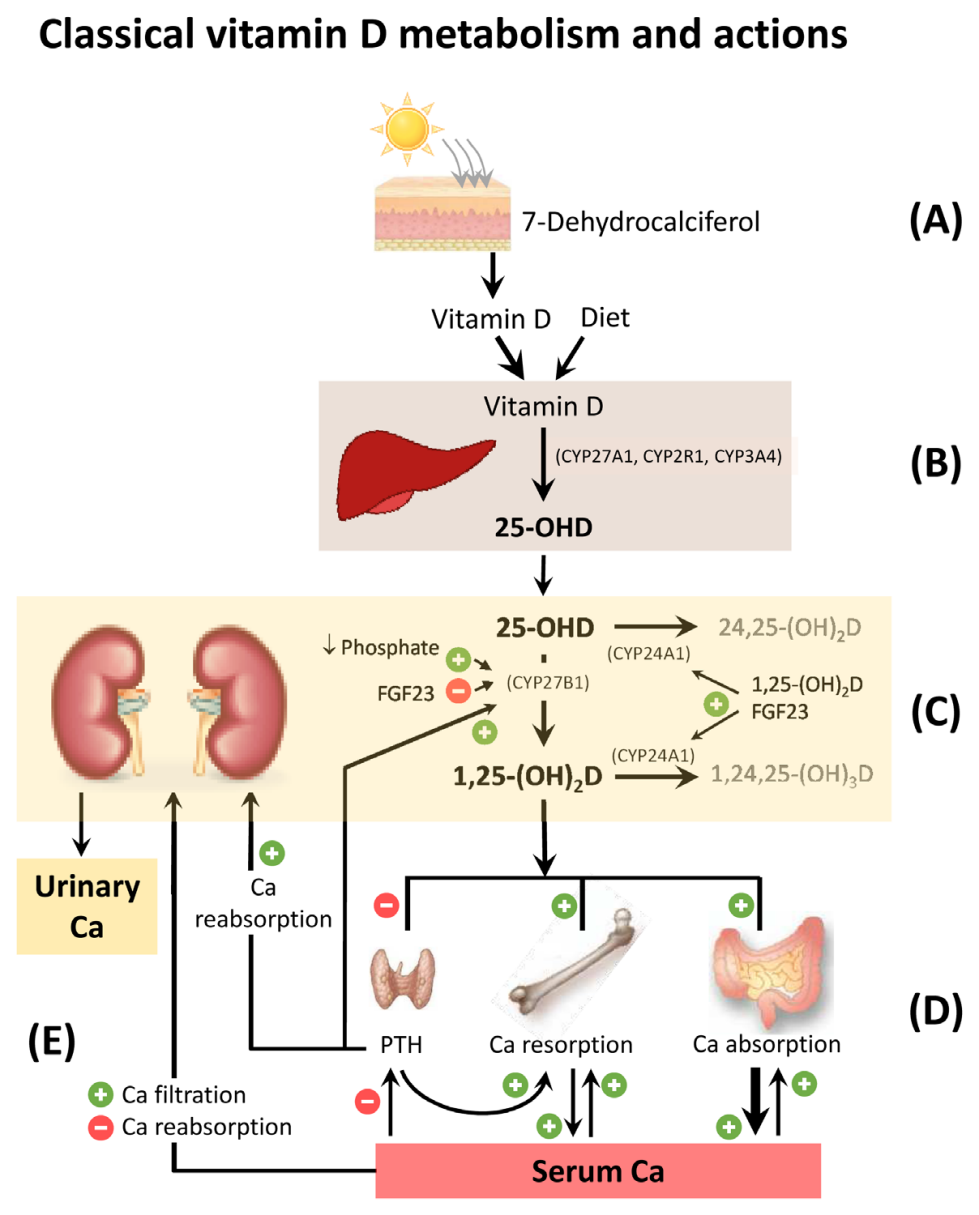
Classical vitamin D metabolism and actions. (**A**) 7-Dehydrocalciferol is transformed by UV light exposure to vitamin D in the skin. (**B**) In the liver, vitamin D is converted to 25-hydroxyvitamin-D (25-(OH)D) by three different 25-hydroxylase enzymes (CYP27A1, CYP2R1, and CYP3A4). (**C**) In the kidney, 25-(OH)D is converted by 25-(OH)D-1-α hydroxylase (CYP27B1) to 1,25-dihydroxyvitamin-D (1,25-(OH)_2_D); this conversion is stimulated by low serum phosphate and high parathyroid hormone (PTH) and inhibited by fibroblast growth factor 23 (FGF23). Both 25-(OH)D and 1,25-(OH)_2_D can be converted to the inactive metabolites 24,25-(OH)_2_D and 1,24,25-(OH)_3_D by the enzyme CYP24A1, which is stimulated by 1,25-(OH)_2_D and FGF23. (**D**) 1,25-(OH)_2_D stimulates both intestinal calcium (Ca) absorption (exceeding excretion) and osteoclastic bone resorption, leading to increased serum Ca concentration. Both 1,25-(OH)_2_D itself and the increased serum Ca inhibit PTH secretion; the lower PTH secretion in turn inhibits renal tubular Ca reabsorption. (**E**) A higher serum Ca increases renal filtered load of Ca and also reduces renal tubular Ca reabsorption. These combined actions result in a net increase in urinary Ca excretion.

Similar to other steroidal hormones, 1,25-(OH)_2_D acts upon a specific intracellular vitamin D receptor (VDR) to fulfill major classical functions on the target organs^41^, including the enhancement of intestinal calcium, phosphate absorption, regulation of osteoclastic bone resorption, and osteoblastic bone formation^42–44^ (Figure 1).

## Extra renal synthesis and non-classical action of 1,25-(OH)_2_D

It has been shown that monocytes, dendritic cells (DCs), macrophages, B-cells, and T-cells possess 25(OH)D-1-α hydroxylase activity locally to transform 25(OH)D into 1,25-(OH)_2_D^45–52^. Similar to its classical action, the immunomodulatory effect of vitamin D (non-classical action) is exerted via the VDR within the immunomodulatory cells^53–59^. This immunomodulatory function plays a key role in the pathogenesis of granulomatous disorders, specifically in sarcoidosis and tuberculosis^11,58^.

Unlike renal (CYP27B1) activity, extrarenal 25(OH)D-1-α hydroxylase CYP27B1 activity is highly substrate dependent, and its production is significantly affected by the prevailing serum concentration of 25(OH)D^60^. Therefore, the local production of 1,25-(OH)_2_D is deficient when serum concentration of 25(OH)D is low (below 25 ng/mL)^60^.

## Vitamin D and innate immunity

**Figure 2.**
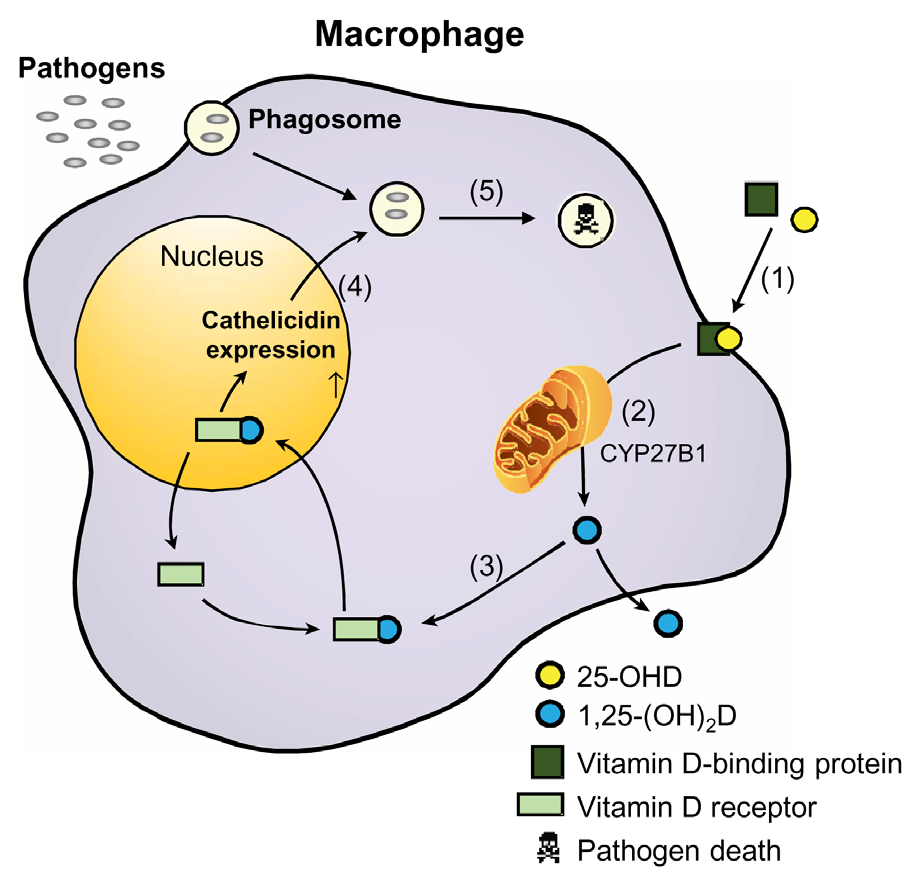
Activation and anti-microbial effects of vitamin D in macrophages. 1) Circulating 25-hydroxyvitamin-D (25-(OH)D) binds to plasma vitamin D-binding protein and enters macrophage. 2) 25-(OH)D is converted into 1,25-dihydroxyvitamin-D (1,25-(OH)_2_D) by mitochondrial CYP27B1. 3) 1,25-(OH)_2_D binds to vitamin D receptor and enters the nucleus. 4) The vitamin D receptor–1,25-(OH)_2_D complex acts as a transcriptional factor, resulting in the expression of cathelicidins. 5) Cathelicidins are incorporated into phagosomes containing internalized pathogens to function as a bactericidal agent.

The effect of vitamin D on the innate immune response is exerted via antigen presentation by macrophages or DCs, the principal cells which mediate the anti-bacterial effect against *Mycobacterium tuberculosis* (M.tb)^61^. The ability of monocytes and macrophages to attack M.tb was dependent not only on M.tb phagocytosis but also on sensing pathogen-associated molecular patterns (PAMPS) via specific pattern-recognition receptors (PRRs) such as Toll-like receptors (TLRs)^62^. TLRs are a family of noncatalytic transmembrane PRRs that interacts with specific PAMPS^63,64^. It was shown that intracrine induction of CYP27B1 and VDR by monocytes follows PAMP sensing by TLRs^65^. This interaction results in the production of cathelicidins, a class of host defense peptides that facilitate microbial killing^61,66,67^ (Figure 2). Granuloma formation may result from defects in innate immunity that impair the elimination of inciting antigens^68^. Since mycobacterial antigens have been implicated in the pathogenesis of sarcoidosis, several studies have suggested that the cathelicidins act as a bridge between sarcoidosis and tuberculosis^69^, and deficiency of cathelicidins in macrophages has been implicated in severe tuberculosis and sarcoidosis^70^. In this study, alveolar macrophage–cathelicidin mRNA expression, VDR, and the VDR coactivator steroid receptor coactivator-3 (SRC3) were measured by quantitative PCR in alveolar macrophages from bronchoalveolar lavage in patients with biopsy-proven sarcoidosis and healthy controls. Results showed reduced alveolar macrophage expression of cathelicidin and SRC3 in severe but not in non-severe sarcoidosis patients compared to controls^70^. Further *in vitro* studies showed that tumor necrosis factor (TNF)-α (a vitamin D3 antagonist) mediates the suppression of SRC3, leading to alveolar macrophage cathelicidin deficiency in severe sarcoidosis.

In a recent prospective case-control study, serum 25(OH)D and cathelicidin levels were measured in 30 patients with active pulmonary tuberculosis, 30 patients with sarcoidosis, and 20 healthy control subjects. Results showed severe vitamin D deficiency in 47% of patients with sarcoidosis compared with 3% in those with tuberculosis^69^. Moreover, cathelicidin levels were significantly higher in control subjects than in sarcoidosis or tuberculosis patients; there was no significant difference in cathelicidin levels between tuberculosis and sarcoidosis patients^69^. An optimum cathelicidin cut-off value of 107.14 pg/mL, with a sensitivity of 81.5% and specificity of 71.2%, was found to differentiate sarcoidosis patients from healthy control subjects.

## Vitamin D and adaptive immunity

Vitamin D action via adaptive immunity may attenuate overzealous inflammatory responses, thus protecting against tissue damage. This action has been demonstrated through downregulation of TLR2 and TLR4 expression in monocytes^71^ and attenuation of T-helper type 1 (Th1) lymphocytes known to increase autoimmune response^64^. The beneficial effects of 1,25-(OH)_2_D are exerted by balancing the proinflammatory cytokines by Th1 cells that produce pro-inflammatory cytokines (including IL-2, IFN-γ, and TNF-α) and Th2 cells that produce anti-inflammatory cytokines (including IL-3, Il-4, IL-5, and IL-10)^61,72–75^ (Figure 2). In addition, two other T-cell groups, Th17 and T-regulatory cells, play a role in the suppression of autoimmunity^76–78^ in response to 1,25-(OH)_2_D. Given the anti-microbial and anti-inflammatory properties of 1,25-(OH)_2_D, it has been suggested that extra renal hydroxylation of 25-OH-D and 1,25-(OH)_2_D represents an adaptive response aimed at minimizing inflammation, protecting against tissue destruction, and eliminating the inciting antigens that cause sarcoidosis^79^.

## Vitamin D in sarcoidosis: a current paradigm

Sarcoidosis and vitamin D deficiency are more common and more severe in AA than in Caucasians in the US^26–28^. Previous studies have shown an inverse association between serum 25-(OH)D and severity of sarcoidosis activity^80–82^. Excess 1,25-(OH)_2_D has been perceived as detrimental, mainly because of concerns for the development of hypercalcemia. However, it is possible that excess production of 1,25-(OH)_2_D in sarcoidosis is an adaptive immune mechanism to mitigate granulomatous inflammation by promoting the removal of inciting antigen to protect tissue integrity. Concern over complications of vitamin D supplementation at the dosage normally insufficient to induce alteration in serum and urinary calcium levels in healthy subjects has hampered research into the role of vitamin D supplementation in sarcoidosis^81,83,84^. However, the incidence of hypercalcemia in sarcoidosis spans a wide range in different populations. In recent studies, the incidence of hypercalcemia ranges from 5.2–7.7% in sarcoidosis patients treated with calcium and vitamin D supplementation^12,80,85^.

Limited studies have addressed mineral status before and after treatment with calcium and vitamin D (Table 1). In 1979, Bell *et al*. implicated 1,25-(OH)_2_D in abnormal calcium metabolism in three patients with sarcoidosis^1^; treatment with prednisone corrected hypercalcemia and normalized serum 1,25-(OH)_2_D. To test whether the development of hypercalcemia is due to the increased sensitivity to vitamin D, seven normal subjects and seven sarcoidosis patients were challenged with oral vitamin D (10,000 IU daily) for 12 days. In this short-term study, vitamin D administration did not change serum calcium, 1,25-(OH)_2_D, or urinary calcium in normal subjects, while serum calcium, 1,25-(OH)_2_D, and urine calcium increased in sarcoidosis patients. The investigators concluded that abnormal calcium metabolism reflects impaired regulation of the synthesis or catabolism of 1,25-(OH)_2_D.

**Table 1.** Studies of vitamin D and Ca supplementation in sarcoidosis.

First author	Mark J. Bolland^85^		Giovanna Capolongo^12^	Lieke S. Kamphuis^80^	Norman H. Bell^1^	
Year	2013		2016	2014	1979	
Design	Randomized, placebo controlled		Non-randomized	Retrospective	Non-randomized	
Number of patients	27		86	301	7	
Type of patients	Normocalcemic sarcoidosis with 25-(OH)D <50 nmol		Sarcoidosis patients with serum 25-(OH)D <75 nmol/L	Sarcoidosis patients	4 normocalcemic patients and 3 patients with history of hypercalcemia	
Age (years)	57		51±6.7	Unknown	22–64	
Female number (%)	19 (70%)		15 (93.7%)	174 (58%)	7 (50%)	
Race/ethnicity	European (77%) Indian (8%) Other (8%)		African American (88%) Caucasian (12%)		Unknown	
Number of patients treated with vitamin D	13		16	104	7	
Intervention						
Type of vitamin D	Cholecalciferol (vitamin D3)		Ergocalciferol (vitamin D2)		Vitamin D2 in propylene glycol was given daily as a single dose	
Dose/frequency/duration	50,000 IU weekly for 4 weeks then monthly for 11 months		50,000 IU once a week for 12 weeks		10,000 IU daily for 12 days	
Diet	Usual diet		Usual diet	Usual Diet	Constant metabolic diet	
Glucocorticoid use	54% past oral use 8% current oral use 46% current inhaled use		48% US patients 47% Italian patients	40% of hypercalcemic patients	Unknown	
Baseline laboratory parameters	Vitamin D (n = 13)	Placebo (n = 14)	Pre-vitamin D (n = 16)	Ca and vitamin D supplementation (n = 104)	Normal subjects (n = 7)	Patients with normal Ca metabolism (n = 4)
25-(OH)D (nmol/L)	40±17	45±17	42±13	46	67.4±14.9	37.4±12.4
1,25-(OH)2D (pmol/L)	109±34	116±25	94±30	114	72.±7.2	70±7.2
Serum phosphorus (mmol/L)	1.23±0.15	1.06±0.17	1.1			
Serum Ca (mmol/L)	2.24±0.06	2.26±0.12	2.38±0.05	2.39	2.37±0.05	2.32±0.05
Parathyroid hormone (pmol/L)	4.0±1.6	4.9±2.0	5.8±3.1			
Urinary Ca (mmol/day)	4.6±3.4	6.6±5.2	3.4±2.3		5.17±0.55	3.42±0.45
Outcome laboratory	Post-vitamin D repletion (n = 13)	Placebo (n = 14)	Post-vitamin D repletion (n = 16)	Ca and vitamin D supplementation (n = 104)	Normal subjects (n = 7)	Sarcoidosis patients with normal Ca metabolism (n = 4)
% hypercalcemia, n (%)	1 (7.6%)	0	1 (6.2%)	5% (4% excluding 1 patient with primary hyperparathyroidism at baseline)	0	0
% hypercalciuria, n (%)	1 (7.6%)	0	2/16 (12.5%)		0	0
25-(OH)D (nmol/L)	80 (68–93)^a^	48 (34–62)^a^	81±25	74	72.4±14.9	69.8±9.98
1,25-(OH)2D (pmol/L)	141 (114–174)^a^	127 (107–140)^a^	49±21		74.4±4.8	79.2±4.8
Serum Ca (mmol/L)	2.24 (2.19–2.30)^a^	2.24 (2.18– 2.29)^a^	2.40±0.15		4.8±0.05	4.8±0.2
Urinary Ca (mmol/day)	7.3 (3.4–11.1)^a^	5.3 (2.6–7.9)^a^	4.2±3.3		4.9±0.45	4.15±0.62

Results are expressed as mean ± SD. ^a^Data are extracted from figures in Boland *et al*. and expressed as mean (95% CI). 25-(OH)D, 25-hydroxyvitamin-D; 1,25-(OH)2D, 1,25-dihydroxyvitamin D; Ca, calcium

Bolland *et al*.^85^, in a randomized, placebo-controlled trial in New Zealand involving 27 normocalcemic patients with sarcoidosis and vitamin D insufficiency using 50,000 IU weekly cholecalciferol for 4 weeks followed by 50,000 IU monthly or placebo for 11 months, showed that vitamin D supplements did not change the mean serum calcium or urine calcium; one patient (7.7%) developed significant hypercalcemia at a cumulative dose of 250,000 IU of cholecalciferol over 6 weeks.

Kamphius *et al*.^80^, in a retrospective study of 301 sarcoidosis patients over 23 years, showed that supplementation of calcium (500 mg) and vitamin D (400 IU) daily was associated with a significant negative correlation between serum 25-(OH)D levels and disease activity assessed by somatostatin receptor scintigraphy; hypercalcemia developed in five out of 104 (4.8%) patients.

**Figure 3.**
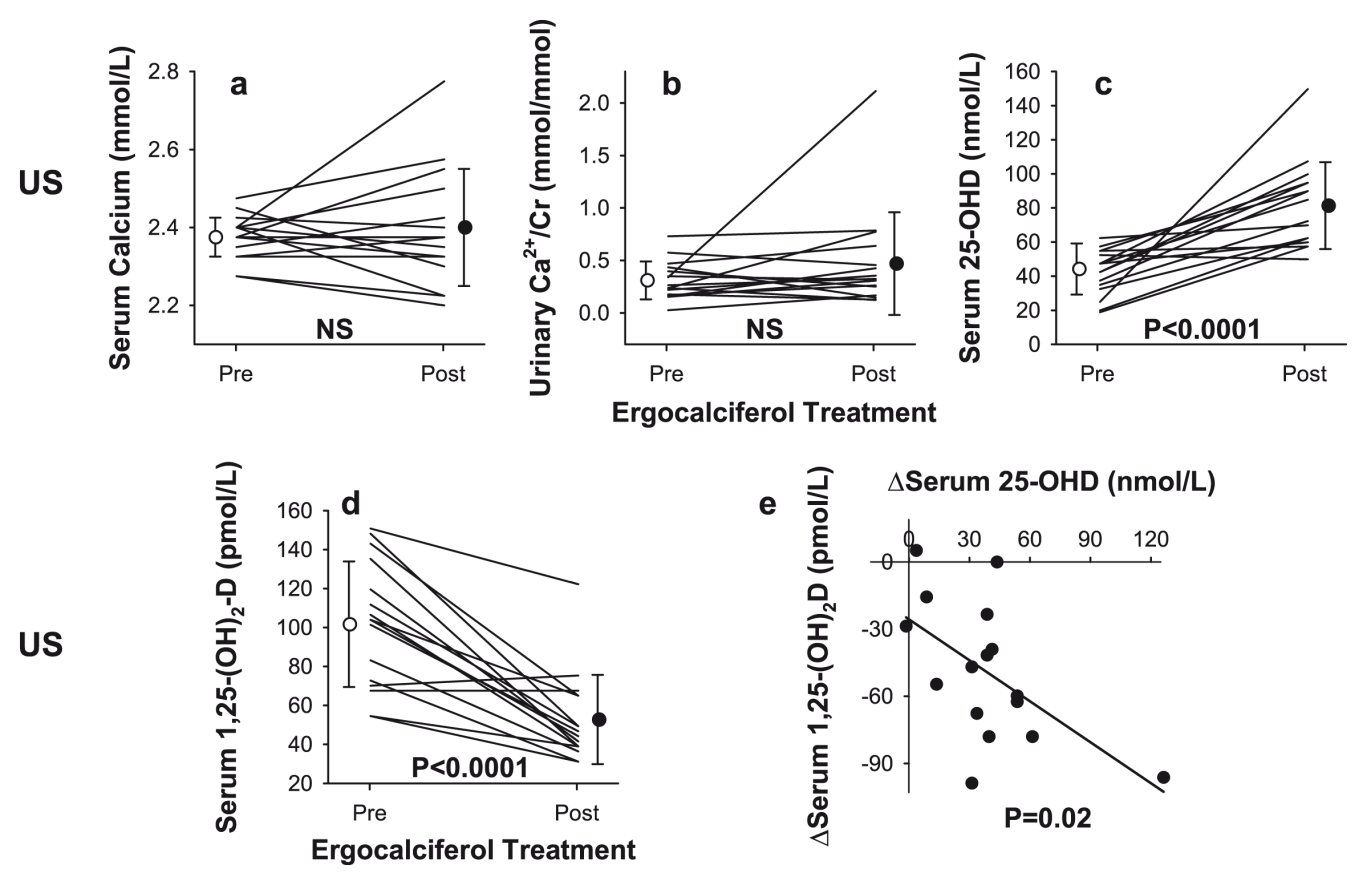
Serum and urine biochemical profiles in patients with sarcoidosis before and after vitamin D supplementation. Changes in (**a**) serum calcium, (**b**) urine calcium, (**c**) serum 25-hydroxyvitamin-D (25-(OH)D), and (**d**) serum 1,25-dihydroxyvitamin-D (1,25-(OH)_2_D) and (**e**) relationship between changes in serum 1,25-(OH)_2_D and 25-(OH)D before and after vitamin D repletion in 16 patients with active sarcoidosis and vitamin D deficiency. This figure was reproduced from Vitamin-D status and mineral metabolism in two ethnic populations with sarcoidosis, Capolongo *et al.*, 64, 1025–34, 2020 with permission from BMJ Publishing Group Ltd^12^.

Capolongo *et al*.^12^ studied two sarcoidosis populations of distinct ethnic and lifestyle backgrounds from the US and Italy and showed largely similar baseline clinical, biochemical, and mineral metabolism parameters. The prevalence of vitamin D insufficiency in patients was not different from that in the correspondingly matched general population^12^. In 16 AA patients with sarcoidosis and vitamin D deficiency, oral ergocalciferol (50,000 IU) weekly for 12 weeks did not alter mean serum calcium level; one patient developed hypercalcemia (6.25%) (Figure 3), i.e. similar to the incidence in Bolland’s study (7.7%)^85^. In both prospective studies^12,85^, the lack of significant changes in bone mineral density or improvement in lung function^12,85^ may be due to the small sample size.

An unanticipated finding in the study by Capolongo *et al*.^12^ is a decline in serum 1,25-(OH)_2_D following vitamin D repletion^12^ (Figure 3). The mechanisms for the decline remain unclear; however, it is possible that, in the presence of vitamin D deficiency, extrarenal production of 1,25-(OH)_2_D in sarcoidosis by immune cells is accentuated as an adaptive response to mitigate antigen-stimulated granuloma formation. While there are no human studies addressing the regulation of serum 1,25-(OH)_2_D in sarcoidosis, a rodent study has shown that locally generated 1,25-(OH)_2_D upregulates 24-hydroxylase, which in turn stimulates the conversion of 25-(OH)D and/or 1,25-(OH)_2_D to the inactive 1,24,25(OH)D^86,87^. However, it has been shown that intracrine stimulation of 24-hydroxylase protein expresses a truncated splice variant which may be functionally inactive. This variant protein maintains its binding and may inhibit the effect of 25-(OH)D, limiting substrate provision for excess 1,25-(OH)_2_D production^88^ spilling over into extracellular space, causing hypercalcemia, but may also mitigate an overzealous autoimmune response^89^. If this hypothesis is validated in a controlled clinical trial, then vitamin D supplementation can be utilized in the treatment of sarcoidosis not only to downregulate the autoimmune response but also to spare steroid-related adverse effects.

## Hypercalciuria and kidney stones in sarcoidosis

**Figure 4.**
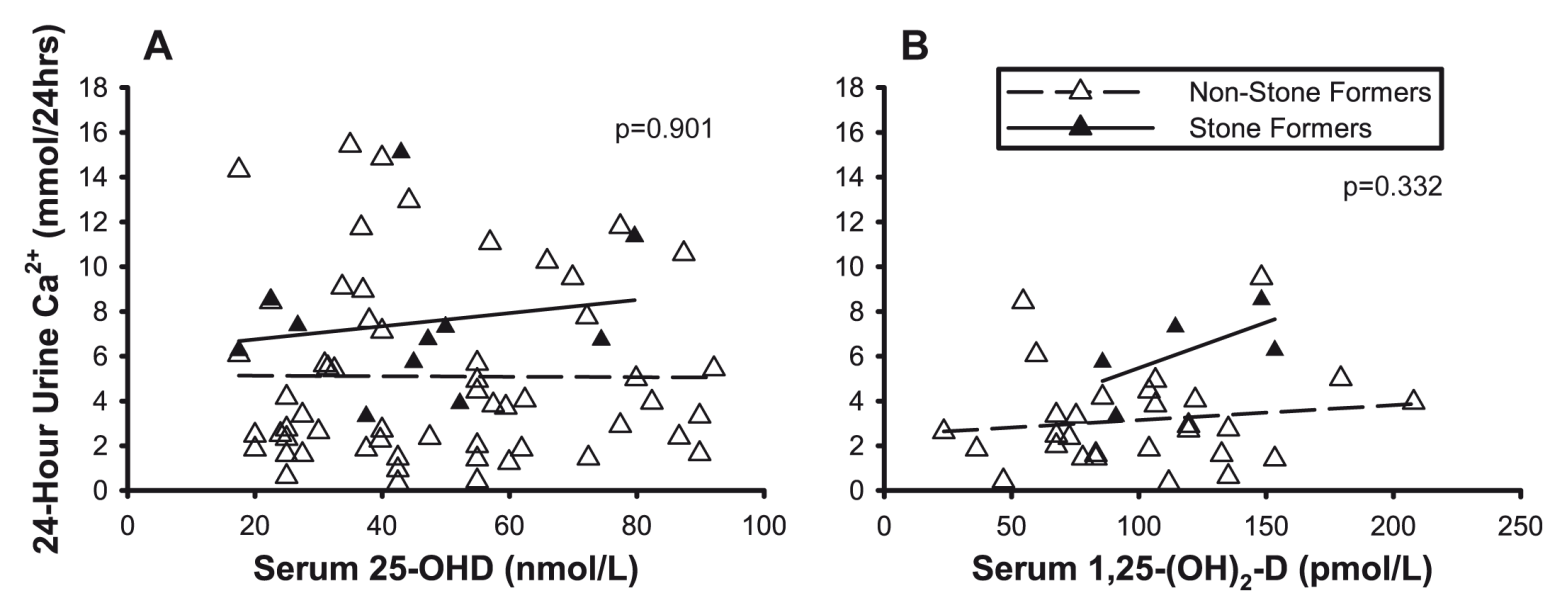
Urinary calcium excretion in non-stone formers and stone-forming subjects with sarcoidosis. Lack of significant relationship of 24 hour urinary calcium with serum 25-hydroxyvitamin-D (25(OH)D) (left panel) and 1,25-dihydroxyvitamin-D (1,25-(OH)_2_D) (right panel) in patients with sarcoidosis. This figure was reproduced from Vitamin-D status and mineral metabolism in two ethnic populations with sarcoidosis, Capolongo *et al.*, 64, 1025–34, 2020 with permission from BMJ Publishing Group Ltd^12^.

Hypercalciuria occurs in 50% of cases of sarcoidosis and increases the risk for calcium oxalate stone formation^90–92^. Approximately 10–13.8% of patients with chronic sarcoidosis suffer from at least one symptomatic stone^93,94^. In only 1% of patients with sarcoidosis, kidney stones are an initial manifestation of the disease^95^. However, in 2.7% of patients, asymptomatic stones are present when sarcoidosis is diagnosed otherwise^96^. In the study by Capolongo *et al*., the prevalence of kidney stones was 11% and 17%, respectively^12^. In this study, kidney stones were associated with a high urinary calcium excretion, but no association was found between serum vitamin D and urinary calcium levels (Figure 4), suggesting that hypercalciuria is independent of serum 25-(OH)D and 1,25-(OH)_2_D levels in this population and may be related to impairment in renal tubular calcium reabsorption as a result of interstitial renal tubule involvement by sarcoidosis^97–100^.

The development of hypercalciuria in sarcoidosis has been attributed to increased intestinal calcium absorption and bone resorption^101,102^. Excess 1,25-(OH)_2_D increases gut calcium absorption^1,103–105^, which increases renal filter load, while decreased serum PTH reduces renal tubular calcium reabsorption^103^. The mechanisms of kidney stone formation in sarcoidosis are similar to those in idiopathic absorptive hypercalciuria because of the increased serum 1,25-(OH)_2_D levels in the latter patients^104,106^. Glucocorticoid treatment produces a significant fall in serum 1,25-(OH)_2_D associated with decreased intestinal calcium absorption in sarcoidosis^103^ but not in absorptive hypercalciuria. Moreover, hypercalciuria in patients with sarcoidosis and high circulating 1,25-(OH)_2_D levels may be due to increased osteoclastic bone resorption^102^. A similar situation has been reported in normal subjects challenged with a large dose of 1,25-(OH)_2_D, suggesting that hypercalciuria originates in part from excessive calcium mobilization from bone^107,108^. In rare instances, nephrocalcinosis due to incomplete renal tubular acidosis may occur in sarcoidosis^109^.

Glucocorticoids are the first-line treatment to reduce endogenous 1,25-(OH)_2_D^110^ and serum calcium levels, often within days. Typically, urinary calcium falls significantly a few days after normalization of serum calcium and 1,25-(OH)_2_D levels in 7–10 days. A lack of response after 2 weeks of treatment should raise the possibility of primary hyperparathyroidism, malignancy, lymphoma, and multiple myeloma. It is customary to reduce glucocorticoid dose within 4–6 weeks of treatment. In case of glucocorticoid failure, hydroxychloroquine^111–113^ or anti-fungal agents such as ketoconazole^114,115^ that inhibit 1-α hydroxylase activity in granuloma may be used as steroid-sparing agents. Immunosuppressive agents such as methotrexate and azathioprine may also be considered^21^. In patients with recurrent kidney stones and persistent hypercalciuria, surgical intervention with shockwave lithotripsy is indicated^116,117^.

## Treatment of abnormal vitamin D metabolism in sarcoidosis

Given that treatment with calcium and vitamin D supplements is required along with the first line of treatment in the management of steroid-induced osteoporosis, this approach was accepted into the 2017 American College of Rheumatology Guideline for the Prevention and Treatment of Glucocorticoid-Induced Osteoporosis^118^. However, the dosage of calcium and vitamin D must be carefully adjusted to avoid the development of hypercalcemia and hypercalciuria. If serum calcium or 24 hour urinary calcium rises above the normal upper limit (2.62 mmol/L) or urinary calcium above 400 mg/day (10 mmol/day) in males and >300 mg/day (7.5 mmol/day) in females, calcium supplements and dietary calcium should be adjusted and serum and urinary calcium levels monitored in 2 weeks. Withdrawal of supplements has been shown to reverse hypercalcemia in sarcoidosis^12,80,81,85,101^. If serum calcium is persistently elevated, then vitamin D dosage must be further adjusted with follow-up blood and urine chemistry. Bolland *et al*.^85^ proposed that given that the nature of hypercalcemia development in this population remains unknown, a smaller dose of vitamin D may avoid complications of hypercalcemia; this issue would need to be examined in future prospective controlled trials. Dose adjustment of vitamin D supplement may reduce its benefits on skeletal health; furthermore, the exact dosage of vitamin D influencing its immunomodulatory role at tissue level has not been tested.

Bolland *et al*., based on modelling, proposed that it is not ethically feasible to conduct a clinical trial aimed at improving skeletal health because the risks of developing hypercalcemia exceed the benefits to bone health. This modelling was based on 13 patients who received vitamin D, in which only one patient developed hypercalcemia. Gallagher *et al*.^119^, in a population of white postmenopausal women with vitamin D insufficiency, found that 8.8% of patients developed hypercalcemia (>2.55 mmol/L), i.e. a prevalence not different from that seen in the small studies of sarcoidosis patients with vitamin D insufficiency following vitamin D/calcium supplementation (4 to 7.6%, Table 1). Therefore, sarcoidosis patients with vitamin D insufficiency do not seem to be at a higher risk of developing hypercalcemia than do other patients commonly administered vitamin D. We believe the benefits of calcium and vitamin D supplementation in sarcoidosis have not been sufficiently examined to determine whether the risk of hypercalcemia outweighs the benefits.

## Conclusion

Vitamin D and calcium disturbances clearly play a principal role in the pathophysiology of sarcoidosis, yet the practical management remains controversial. Because of the concerns of worsening abnormal calcium metabolism following vitamin D supplementation, the clinical community has been ambivalent on supplementation in vitamin D-deficient or -insufficient patients with sarcoidosis. This concern also limited the conduct of prospective clinical trials to address a novel but neglected aspect of vitamin D action in this population. A study in two distinct ethnic groups of patients with sarcoidosis has opened the door towards further unraveling the role of vitamin D^12^. The result of the study showing that repletion of 25-(OH)D may reverse some underlying pathophysiological abnormalities was compelling; the associated lowering of serum angiotensin-converting enzyme (ACE) and serum γ-globulin, both surrogate markers of active sarcoidosis, supports the suppression of granulomatous immune activity. These intervention studies were small in size and did not allow comprehensive investigation of the potential risk–benefit balance of vitamin D supplementation on different organ systems. Further prospective interventional investigation involving larger cohorts of patients is warranted to clarify the relationship between vitamin D repletion and inflammatory activity and outcome in sarcoidosis.
